# Colorectal Cancer: A Brief and Simplified Analysis of a Complex Disease

**DOI:** 10.3390/medicina60122034

**Published:** 2024-12-10

**Authors:** Krasimir Petrov, Ivan Ivanov, Savelina Popovska, Tatyana Betova, Zornitsa Kamburova

**Affiliations:** 1 Department of Pathology, Faculty of Medicine, Medical University, 5800 Pleven, Bulgaria; 2 Department of General and Clinical Pathology, University Multi-profile Hospital for Active Treatment “Georgi Stranski”, 5800 Pleven, Bulgaria; 3 Department of Medical Genetics, Faculty of Pharmacy, Medical University, 5800 Pleven, Bulgaria

**Keywords:** colorectal adenocarcinoma, left and right-sided, histologic type, peritumoral budding, lymphovascular and perineural invasion, lymph node metastasis, MMR status, mutations, NGS

## Abstract

*Background and Objectives:* This study examined factors influencing the onset and progression of colorectal tumors, including patients’ epidemiological data, tumor location (right-sided, left-sided, and rectal), histomorphology, perineural or intraneural invasion, lymph node status, immune reactions, mismatch repair (MMR) status, and commonly observed mutations. Our primary goal was to evaluate their predictive and prognostic value and interactions. *Materials and Methods:* We analyzed a retrospective cohort of 100 patients with colorectal adenocarcinoma diagnosed between 2020 and 2023, using formalin-fixed paraffin-embedded (FFPE) tumor blocks. The methods included routine H&E microscopy, immunohistochemistry, Next-Generation Sequencing (NGS), and subsequent statistical analysis. *Results:* The findings showed a median diagnosis age of 70 years, with no gender-specific tumor localization. Right-sided tumors were prevalent, especially among patients with a defective MMR (dMMR), which represented 89% of dMMR cases. MMR status significantly correlated with tumor localization. We observed significant relationships between tumor grade, lymphovascular invasion, and overall tumor stage. Higher tumor grades and stages correlated with increased lymphovascular invasion and lymph node involvement. Interestingly, tumor budding did not correlate with lymph node metastasis but was significantly associated with higher tumor grades. Most BRAF mutations were found in right-sided tumors, indicating a significant correlation with this localization. *Conclusions:* This study focuses on the diversity of colorectal cancer (CRC) by examining how genetic and histological characteristics vary based on tumor location or other tumor variables.

## 1. Introduction

Cancers of the colon and rectum are among the leading causes of illness and death worldwide, contributing significantly to the global disease burden, with rising incidence rates in many regions.

According to data from 2022, globally, for both sexes, colon carcinomas rank fourth in incidence, with approximately 1,150,000 cases annually, and fifth in mortality, with approximately 540,000 cases. Rectal carcinomas, on the other hand, are eighth in incidence, with approximately 740,000 cases, and tenth in mortality, with approximately 340,000 deaths per year for the same period [1].

In the United States, it is estimated that there will be 152,810 new cases of colon and rectal cancers combined for both sexes. Additionally, the estimated number of deaths from these cancers in both sexes will be 53,010 in 2024. Colorectal cancer has now become a leading cause of death in men and the second leading cause in women under the age of 50 [2].

Regarding Europe, the data from the ECIS-European Cancer Information System for 2022 estimate that, for both sexes, colorectal carcinomas rank second in incidence with 448,619 cases and also second in mortality with 203,482 cases.

In Bulgaria, the estimated number of new cases of colorectal cancer was reported to be 5096 in 2022, with an estimated 2079 deaths, making it the second most common cause of cancer mortality for both sexes [3].

Based on data from the Global Cancer Observatory (International Agency for Research on Cancer), the estimated number of new cases of colorectal cancer is projected to increase by approximately 61% between 2022 and 2045. Similarly, the number of deaths from colorectal cancer is expected to rise by approximately 77% during the same period [4].

Recent understanding of colorectal carcinoma reveals that its etiology and development are influenced by a multitude of factors, such as diet [5,6,7], lifestyle habits [8,9,10], social and economic status [11,12], and environmental exposures, some of which can be modified and can be addressed through prevention strategies. However, other factors, such as driver gene mutations and certain genetic predispositions, are not modifiable, highlighting the complexity of the disease [13,14].

Research indicates that there are sex-specific differences in several factors related to the disease, including predisposing conditions, tumor location, and mutational profile. These differences may stem from complex regulatory mechanisms associated with the X chromosome, variations in how sex hormones influence the immune response, and the distinct characteristics of the microbiome in males and females. However, findings show that there are no significant differences in the age at which the disease is diagnosed between the sexes. Limited studies with a small sample size suggest notable variations in toxicity associated with conventional treatment methods such as 5-fluorouracil, as well as differences in therapeutic outcomes when utilizing targeted therapy and immunotherapy [15,16,17].

In 1990, Bufill analyzed colorectal carcinomas from different anatomical locations and proposed that their characteristics vary depending on whether they are located proximally (right side) or distally (left side). His hypothesis is based on the differences in the embryonic origins of the right colon (midgut) and the left colon (hindgut) [18].

Since this hypothesis was proposed, numerous studies have confirmed the presence of characteristics that depend on the location of the primary tumor—whether it is in the right colon, left colon, or rectum—and demonstrate that there are location-dependent variations in several indicators, including epidemiological aspects (such as age and sex) and tumor characteristics (such as histological subtype, mutations, and molecular features). Additionally, these studies show differences in response to various treatment regimens and variations in patient survival rates [19,20,21,22,23,24].

Tumor morphology is another focus of various researchers’ studies. Their results indicate that specific histological subtypes, such as signet ring, mucinous, and poorly differentiated tumors, are associated with a significantly higher risk of recurrence and overall mortality [25,26,27,28].

Various tumor characteristics, such as lymphatic and vascular invasion and perineural and intraneural invasion, are important prognostic factors and risk factors for lymph node metastases, poor prognosis, local and distant recurrences, and decreased survival in patients with colorectal cancer [29,30,31].

Another prognostic factor for patients with colorectal cancer is the immune response. Research indicates that the presence of intratumoral or peritumoral lymphocytes, as well as the “Crohn-like” immune reaction, is associated with a more favorable prognosis [32,33,34].

Epithelial–mesenchymal transition is a process observed at the invasive front of tumors and is referred to as tumor budding. This phenomenon can be classified into two types: peritumoral budding, which occurs at the tumor’s invasive front, and intratumoral budding, which takes place within the tumor itself. During the epithelial-mesenchymal transition, epithelial cells transform into cells with mesenchymal characteristics, enhancing the tumors’ invasive potential. This transition is also a significant factor contributing to therapy resistance and serves as a poor prognostic indicator, particularly in the case of colorectal carcinomas [35,36,37,38,39,40,41].

Predictive biomarkers in colorectal carcinoma, such as mismatch repair (MMR) status and the mutational tumor profile—including BRAF, KRAS, NRAS, TP53, PIK3CA, and other mutations—carry biological and therapeutic significance. Mutations in the KRAS and NRAS genes are associated with resistance to monoclonal antibodies. In contrast, a defective mismatch repair mechanism is crucial for oncogenesis and serves as a vital test for diagnosing Lynch syndrome.

Deficient mismatch repair (dMMR) or microsatellite instability generally indicates a favorable prognosis, especially in the absence of BRAF gene mutations. Conversely, proficient mismatch repair (pMMR) or microsatellite-stable tumors that harbor BRAF mutations are considered poor prognostic factors. Additionally, dMMR/microsatellite unstable tumors often show resistance to 5-fluorouracil therapy but tend to respond well to PD-L1 inhibitor therapy [28,42,43].

Our study aimed to investigate and establish tumors’ epidemiological, locational, and morphological characteristics in a selected cohort of patients. We also examined various predictive and prognostic factors related to colorectal carcinoma and assessed the presence or absence of interactions among them. The indicators we focused on include the following:

- Patients’ epidemiological data;
- Primary tumor location—right-sided, left-sided, and rectal tumors;
- Tumor histomorphology;
- Perineural or intraneural invasion;
- Lymph node status;
- Peritumoral stromal immune reaction;
- Peritumoral budding;
- MMR status;
- Some of the frequently observed mutations in colorectal carcinomas.

## 2. Materials and Methods

This study was conducted retrospectively, with 100 patients diagnosed with colorectal adenocarcinoma between 2020 and 2023. These patients were selected from the Department of Clinical Pathology archives at “Georgi Stranski” University Hospital in Pleven, Bulgaria.

### 2.1. Patient Selection

#### 2.1.1. Inclusion Criteria

In selecting patients for the study, we adhered to the following inclusion criteria:

- Patients who provided informed consent to participate in the study;
- Patients who had undergone resection of the colon or rectum for primary adenocarcinoma.

The selection was carried out randomly. A small group of patients who had received preoperative radiation or chemotherapy was intentionally not excluded.

#### 2.1.2. Exclusion Criteria

- Presence of other histological tumor types: pure neuroendocrine tumors/carcinomas, benign tumors, mesenchymal tumors, or metastatic tumors of the colon or rectum;
- Patients who declined to provide informed consent for participation.

This study was approved by the Ethics Committee of Medical University, Pleven, before its commencement.

### 2.2. Methods

#### 2.2.1. Assessment of Peritumoral Budding

We used biopsy samples fixed in 10% buffered formalin, embedded in paraffin, cut at 4 µm, and stained with H&E.

Tumor buds were counted on H&E-stained slides according to the algorithm described by the International Tumor Budding Consensus Conference [37]. We also encountered cases with zero budding during the evaluation, which were included in the Bd1 group [44].

Ten fields were scanned using a 10× objective lensto identify the invasive front of the tumor and the TB hotspot area.

PTB was counted in the hotspot area using a 20× objective lens, and the result was divided by the normalization factor to calculate the TB count at 0.785 mm² (in our study, the normalization factor was 0.810).

Tumor budding was graded as follows:

Bd1: Low—0–4 tumor buds;
Bd2: Intermediate—5–9 tumor buds;
Bd3: High—10 or more tumor buds.

#### 2.2.2. Assessment of Tumor MMR Status

IHC procedure:

Tumor resection specimens from patients with colorectal adenocarcinoma were fixed in 10% buffered formalin for 24 to 36 h at room temperature, dissected, and embedded in paraffin. A pathologist selected 5 µm thick parallel sections of representative invasive tumor tissue and normal mucosa, confirmed by the H&E-stained slides.

Epitope retrieval was performed for 20 min at 97 °C using DAKO PT Link (code PT100/PT101).

The tumor sections were stained with the following ready-to-use antibodies (DAKO Denmark©, lostrup, Denmark):

MLH1: ES05 (Monoclonal mouse Anti-Human MutL Protein Homolog 1 (ready to use; Ref. No. IR079).

MSH2: FE11 (Monoclonal mouse Anti-Human MutS Protein Homolog 2 (ready to use; Ref. No. IR085).

MSH6: EP49 (Monoclonal rabbit Anti-Human MutS Protein Homolog 6 (ready to use; Ref. No. IR086).

PMS2: EP51 (Monoclonal rabbit Anti-Human Postmeiotic Segregation Increased 2 (ready to use; Ref. No. IR087).

EnVision FLEX (K800021-2), High pH (Link), HRP. Rabbit/Mouse. A high-pH Immunohistochemistry Visualization system kit was used.

The incubation time for all antibodies was 20 min at room temperature.

A Dako Agilent Autostainer Link 48 slide stainer was used to perform the staining. External reagent negative controls were prepared as part of the manufacturer’s reagent kit. Normal colonic mucosa, stromal cells, and stromal lymphocytes from the same slides containing the tumor were used as internal positive controls.

Results analysis:

A pathologist analyzed the results. The expression was categorized as follows:

Normal (retained): nuclear expression present in >10% of tumor cells, along with retained expression in internal controls.

Negative (loss): absence of nuclear expression in tumor cells, with retained expression in internal controls.

In our study, we did not aim to evaluate the subclonal expression of MMR proteins or use immunohistochemistry for the confirmation of peritumoral budding.

#### 2.2.3. Investigation of Lymphovascular Invasion—LVI

Due to the relatively small sample size and the limited number of colorectal cancer patients with observed lymphatic and vascular invasion, we did not aim to analyze these two morphological characteristics separately. Additionally, we did not focus on distinguishing between intramural and extramural vascular invasion, despite existing evidence indicating their negative prognostic significance [29,30,45].

#### 2.2.4. Assessment of Perineural Invasion—PNI

The assessment of perineural invasion (PNI) is based on the identification of tumor cells within any of the three layers of the nerve sheath, following the definition by Liebig C. et al. [31].

#### 2.2.5. Assessment of Immune Response

In our work, we focused on evaluating the peritumoral stromal inflammatory reaction at the invasive tumor front, specifically assessing the immune response of the so-called “Crohn-like” type. For practical purposes, we conducted a semi-quantitative assessment of the peritumoral stromal inflammatory reaction, categorizing it into three levels using Graham and Appelman’s method: marked—3 or more lymphoid aggregates; moderate—the presence of 2 or fewer lymphoid aggregates; and absent—without lymphoid aggregates [32,46,47].

#### 2.2.6. NGS Testing

Genetic testing of tumor tissues was performed using NGS, with the TruSight Tumor 15 panel (Illumina. Inc., San Diego, CA 92122, USA). Representative tumor tissue blocks, fixed in 10% neutral buffered formalin and embedded in paraffin (FFPE), were selected and cut into 5 µm sections, ensuring sufficient tumor content for analysis. DNA extraction, quality and quantity assessment, and library preparation were carried out according to the manufacturer’s protocols. The sequencing platform used was Illumina NextSeq 550 (Illumina. Inc., San Diego, CA 92122, USA), following the manufacturer’s guidelines. Data analysis and result interpretation were performed by qualified medical bioinformatics and medical genetics specialists.

#### 2.2.7. Statistical Analysis

The results and other data were recorded and analyzed using IBM SPSS Statistics 24.0. The chi-square test was used to compare categorical data, with values of *p* < 0.05 considered statistically significant. The Kruskal–Wallis test was employed for parametric data if they did not follow a normal distribution. We used normalization to adjust the data within a specific range, ensuring that features or variables with different scales did not disproportionately influence the results of the analyses.

## 3. Results

### 3.1. Epidemiological Data

#### 3.1.1. Sex and Age

As mentioned above, a total of 100 patients were examined, with the following epidemiological characteristics: 43 females and 57 males, ages ranging from 48 to 83 for females and 45 to 88 for males, with a median age of 70 for both sexes.

#### 3.1.2. Sex and Tumor Localization

**Figure 1:**
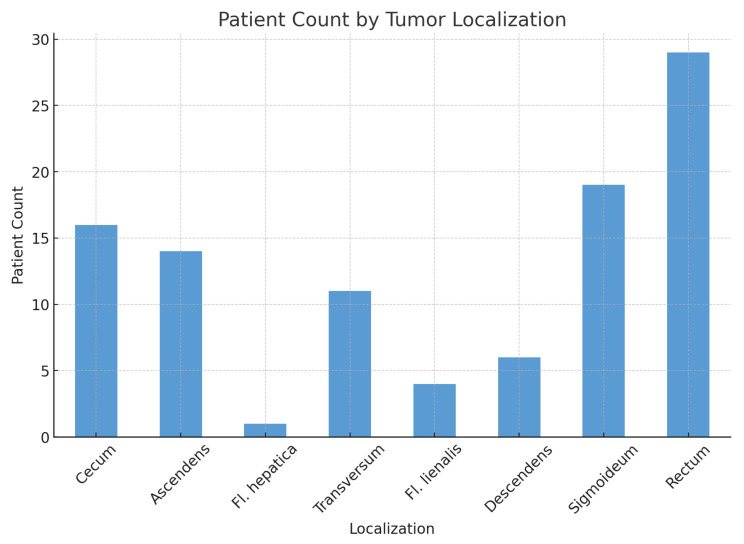
Tumor localization distribution for the whole patient group (patient count).

Tumor localization across different anatomical sites for the entire patient group is presented in Figure 1. The analysis shows no statistically significant association between tumor localization and sex ($\chi^{2}$: 2.95; *p*-value: 0.89; Df: 7).

**Figure 2:**
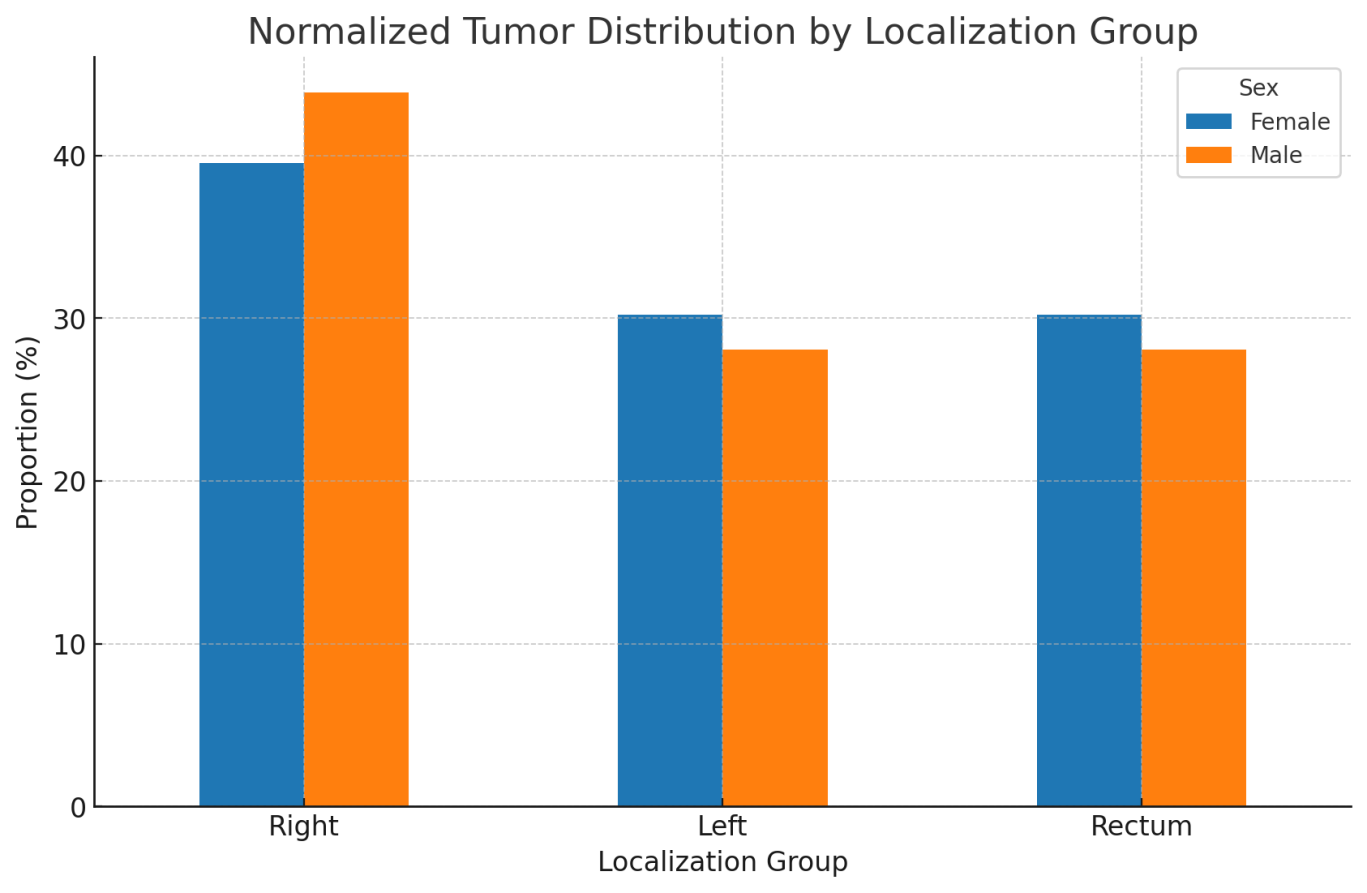
Normalized simplified tumor localization groups: right colon, left colon, and rectum.

After consolidating the tumor localizations, we formed groups of patients with left-sided, right-sided, and rectal localization of the primary tumor. After normalization, we obtained the results presented in Figure 2.

### 3.2. Data on Tumor Characteristics

The results regarding some characteristics of the primary tumor in the studied patient group are as follows and presented in Table 1:

#### 3.2.1. Primary Tumor Localization and Dependencies with Subsequent Parameters

- Age

Based on the different embryological origins of the right side of the colon (cecum, ascending colon, hepatic flexure, and the proximal two-thirds of the transverse colon) and its left side (splenic flexure, descending colon, and sigmoid colon), as well as extensive experimental and clinical data on differences in morphology, genetics, and clinical characteristics, we consolidated the primary tumor localizations into three groups: right colon, left colon, and rectum. The subsequent statistical analysis showed no statistically significant differences regarding the primary tumor localization, age, or sex of the patients.

The results of the Kruskal–Wallis tests are as follows: Females—the test statistic is 0.805 with a *p*-value of 0.669. Males—the test statistic is 0.418 with a *p*-value of 0.811.

- Histological subtype

In the analysis of grouped histological subtypes for each localization category (right colon, left colon, and rectum), the results indicate that there is no statistically significant relationship between localization and histological subtype: ($\chi^{2}$: 19.937; *p*-value: 0.336; Df = 18). Additionally, when the statistical results of the chi-square test are examined by sex, no significant differences are observed: males—*p*-value: 0.685; females—*p*-value: 0.380.

- Tumor Grade and tumor Stage

The potential relationships between primary tumor localization (right colon, left colon, and rectum) and tumor Grade and Stage were examined. The results from the chi-square tests indicated that there are no statistically significant differences among these groups. Furthermore, when the analysis was conducted separately by sex, no significant differences were found in either tumor grade or stage. Specifically, the results for tumor grade were as follows: Males—*p*-value: 0.429; Df = 6. Females—*p*-value: 0.096; Df = 4. The results for tumor stage were as follows: Males—*p*-value: 0.230; Df = 8. Females—*p*-value: 0.154; Df = 10. (Note: the ypT categories were added to their respective T groups).

**Figure 3:**
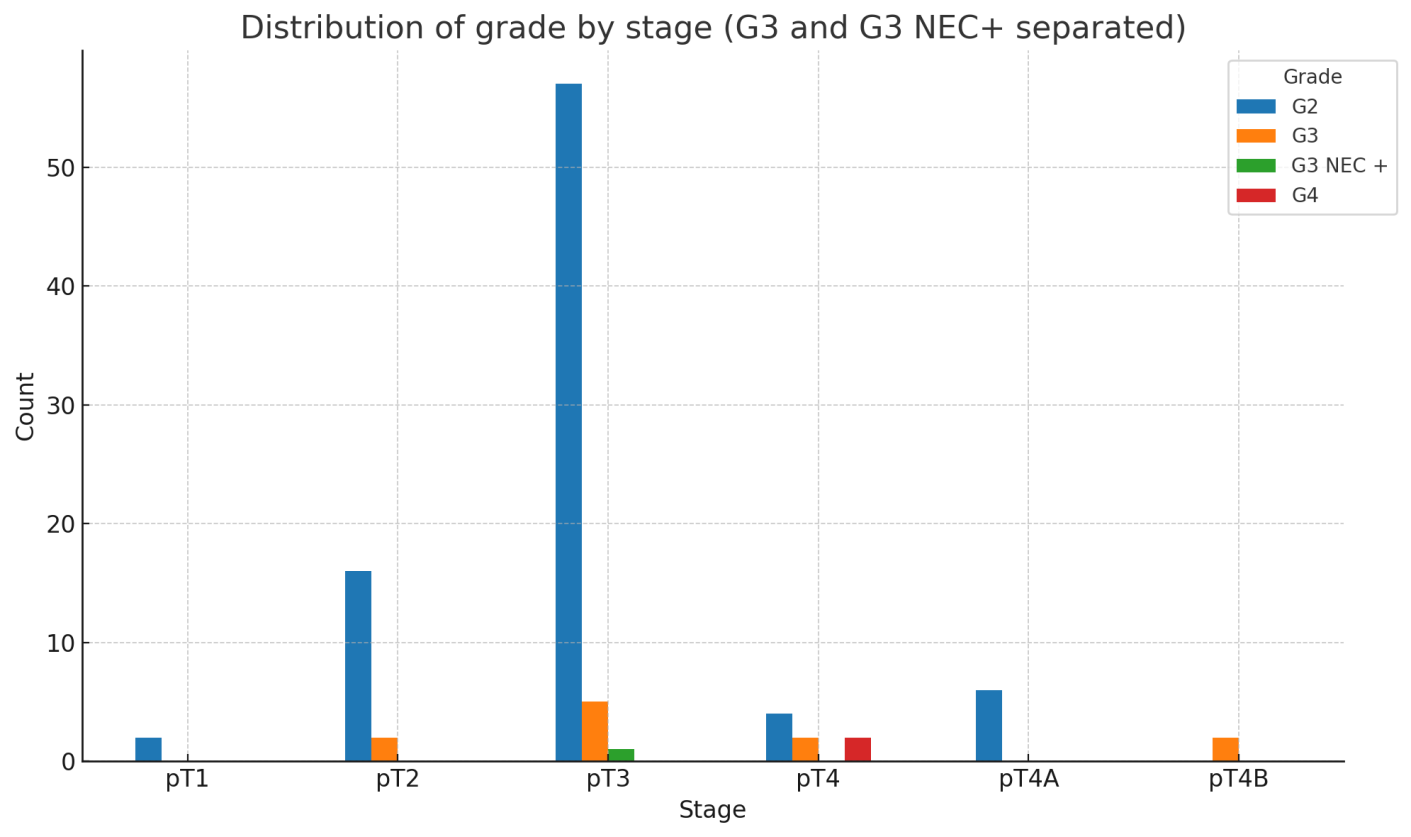
Proportional distribution of G2, G3, and G4 within each stage.

However, when we compare tumor differentiation (G) and tumor stage (pT), the following expected results are obtained (Figure 3).

As expected, the chi-square test shows that the relationship between grade and stage is highly statistically significant ($\chi^{2}$: 42.69; *p*-value: 0.00000565; Df = 10).

- Lymph node status

The statistical analysis of the patient samples showed no statistically significant differences in the N status of patients with colorectal adenocarcinoma based on the localization of the primary tumor—right colon, left colon, or rectum—for the entire patient group ($\chi^{2}$: 11.811; *p*-value: 0.621; Df = 14). The same conclusions remain valid when analyzed separately by sex: Males—$\chi^{2}$: 12.82; *p*-value: 0.382; Df = 12. Females—$\chi^{2}$: 8.38; *p*-value: 0.869; Df = 14.

When we compared lymph node status with the degree of tumor differentiation and tumor stage, the results indicated a lack of statistically significant differences once again, as is most clearly seen from the statistical analysis: Grade—$\chi^{2}$: 16.945; *p*-value: 0.259; Df = 14. Stage—$\chi^{2}$: 36.590; *p*-value: 0.395; Df = 35.

#### 3.2.2. Important Histological Features of the Tumor

Lymphatic and vascular invasion:

In our study, we did not intend to analyze these two morphological characteristics separately due to the relatively small sample size. Although evidence suggests their negative prognostic significance, we did not focus on differentiating between intramural and extramural vascular invasion. Therefore, we address both characteristics together in the following brief section.

**Figure 4:**
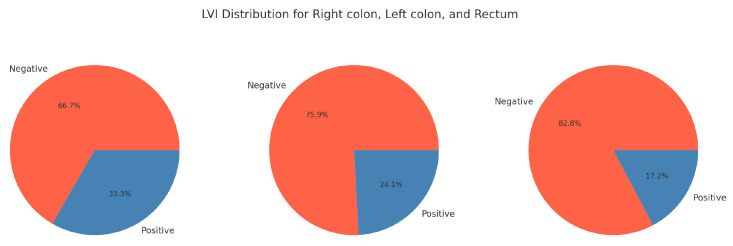
LVI distribution for right colon, left colon, and rectum.

- LVI and primary tumor localization:

Figure 4 visually presents the relationships between the localization of the primary tumor and lymphovascular invasion (LVI). The chi-square test results for the association between LVI status and localization are as follows: chi-square value: 2.38; Df: 2; *p*-value: 0.304. The *p*-value is greater than 0.05, indicating that there is no statistically significant association between LVI status and localization.

When comparing the lymph node statuses across different localization groups—right colon, left colon, and rectum—we find the following results regarding the number of patients with negative or positive lymph nodes:

- Right colon: negative—28; positive—14.
- Left colon: negative—22; positive—7.
- Rectum: negative—24, positive—5.

- LVI and histopathological subtype:

The statistical analysis of the proportions of negative and positive statuses across various tumor histological subtypes shows no statistically significant association between lymphovascular invasion (LVI) and histological subtype. The Monte Carlo approximation of the chi-square test for the relationship between LVI status and histological subtype produced the following results: chi-square value—15.93; Df—9; Monte Carlo *p*-value—0.068. However, the *p*-value is close to significance, suggesting a potential trend that may warrant further investigation.

- LVI and tumor Grade and Stage:

As we expected, a statistically significant association exists between lymphovascular invasion and tumor grade and stage. The results of the chi-square tests are shown in Table 2.

Perineural invasion:

The assessment of perineural tumor invasion is based on the presence of tumor cells in any of the three layers of the nerves or tumor cells involving at least one-third of the nerve’s circumference. We observed the following results regarding this tumor characteristic, as shown in Table 3. There are no statistically significant associations between PNI and any of the variables (primary tumor localization, tumor histological subtype, tumor grade, and tumor stage).

##### Immune Response

**Figure 5:**
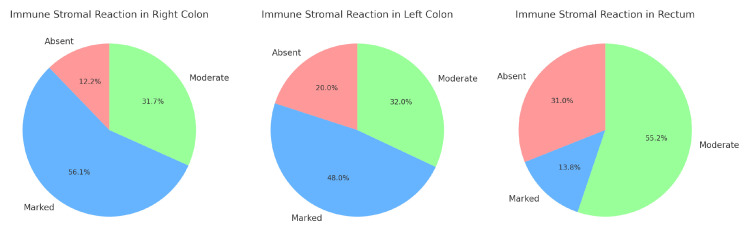
Immune stromal reaction and primary tumor localization.

As mentioned above, we assessed the immune response associated with the “Crohn-like” type. Our focus is to examine potential interactions between this immune reaction and various prognostic factors, including tumor budding, MMR status, mutational status, and the localization of the primary tumor. After data processing, we obtained the following results:

Immune response and primary tumor localization—Figure 5:

The right colon has the highest percentage of marked reactions (56.1%). In contrast, absent reactions are relatively low.

The left colon shows a more balanced distribution, with the majority of cases presenting marked reactions (48%).

The rectum is unique in having the highest percentage of moderate reactions (55.2%) and the lowest percentage of marked reactions (13.8%).

Results from the chi-square test—$\chi^{2}$ value: 14.05; Df: 4; *p*-value: 0.007. The *p*-value suggests that there is a strong indication that the distribution of peritumoral stromal immune reactions is not uniform across the three tumor localizations (right colon, left colon, and rectum). In other words, the type or intensity of the immune response differs significantly depending on the tumor’s primary location.

**Figure 6:**
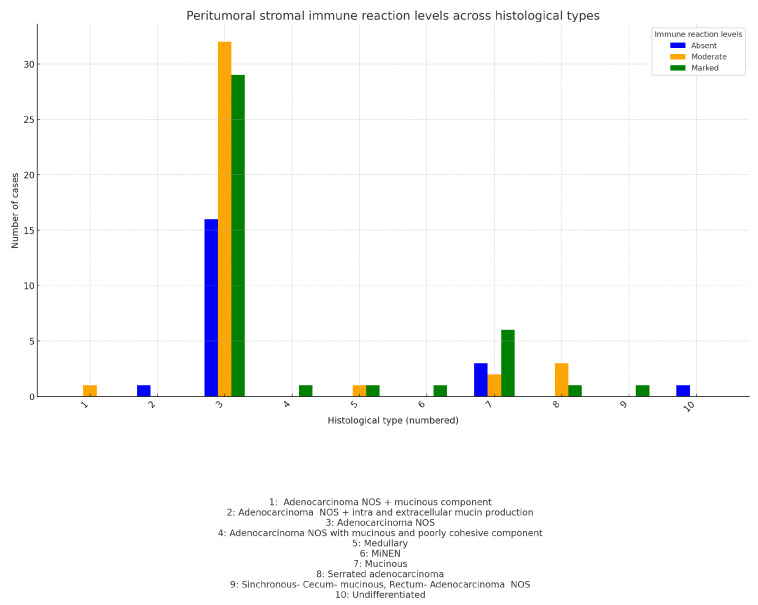
Peritumoral stromal reaction by histological subtype.

Immune response and histological subtype:

The chart compares each histological subtype’s average peritumoral stromal reaction. Each point represents the average immune response (from absent to marked) within each histological subtype (Figure 6):

As shown in the chart, adenocarcinoma NOS and adenocarcinoma NOS with an additional tumor component (such as synchronous: cecum(mucinous) and rectum (adenocarcinoma NOS); adenocarcinoma NOS and adenocarcinoma with intra- and extracellular mucin production; or MiNEN with an exception) exhibit a marked immune response.

The following are the results from the chi-square test: $\chi^{2}$ value: 18.76; *p*-value: 0.4067; Df: 18. These results show no statistically significant association between histological subtype and the peritumoral stromal reaction. This suggests that while some histological subtypes may show a pronounced immune response, there is not a consistent or significant pattern across the studied histological subtypes.

Immune response and tumor grade and stage:

The relationships between tumor grade, tumor stage, and peritumoral stromal immune reaction were evaluated. The results for both characteristics showed no statistically significant findings, indicating a lack of strong association between tumor grade or stage and the intensity of the peritumoral stromal immune reaction.

1. Tumor grade (G1, G2, G3) vs. peritumoral stromal immune reaction—chi-square value ($\chi^{2}$): 0.185; *p*-value: 0.996; Df: 4.
2. Tumor stage (T1, T2, T3, T4) vs. peritumoral stromal immune reaction—chi-square value ($\chi^{2}$): 0.685; *p*-value: 0.995; Df: 6.

These findings suggest that the immune response in the tumor’s surrounding stroma does not significantly correlate with either the differentiation (grade) or progression (stage) of the tumor in this specific cohort.

Immune response and lymph node status:

The chi-square test results for the relationship between peritumoral stromal immune reaction and lymph node status are as follows:

Chi-square value: 14.47; *p*-value: 0.416; Df: 14.

These results indicate no significant association between peritumoral stromal immune reaction and lymph node status. This suggests that variations in the immune reaction in the tumor stroma do not correlate significantly with whether lymph node involvement is present or not.

Immune response and lymphovascular (LVI) and perineural invasion (PNI):

The results of the chi-square tests show no statistically significant association between these three parameters.

1. Peritumoral stromal immune reaction vs. lymphovascular invasion (LVI): chi-square value: 2.23; *p*-value: 0.327; Df: 2.
2. Peritumoral stromal immune reaction vs. perineural invasion (PNI): chi-square value: 3.08; *p*-value: 0.214; Df: 2.

The lack of a significant correlation suggests that the peritumoral stromal immune reaction does not directly influence lymphovascular or perineural invasion in the context of the cases studied.

##### Tumor Budding

In our study, when assessing tumor budding, we followed the classical scheme described by the International Tumor Budding Consensus Conference (ITBCC). We also encountered cases with zero budding during the evaluation, which were included in the Bd1 group. After evaluating the tumor budding, we obtained the following results:

Peritumoral budding and primary tumor localization:

The statistical analysis results showed no statistically significant differences among the groups for peritumoral budding and primary tumor localization (right colon, left colon, and rectum) and for peritumoral budding vs. histological subtype.

Peritumoral budding and tumor grade and stage:

The results for peritumoral budding grades and tumor grade and stage are shown in Table 4.

The chi-square test results for the association between peritumoral budding and both tumor grade and tumor stage are as follows:

1. Tumor grade vs. peritumoral budding: Chi-square value: 14.39; *p*-value: 0.0255; Df: 6. This result indicates a statistically significant association between peritumoral budding and tumor grade.
2. Tumor stage vs. peritumoral budding: The result suggests no statistically significant association between peritumoral budding and tumor stage.

Peritumoral budding and lymphovascular invasion (LVI) and lymph node status:

The analysis of the association between peritumoral budding and lymphovascular invasion indicates a statistically significant correlation. The results from the chi-square test are as follows: chi-square value: 8.59; *p*-value: 0.0137; Df: 2. Specifically, higher levels of budding may be linked to an increased likelihood of lymphovascular spread in colorectal cancer. This finding underscores the importance of evaluating both peritumoral budding and lymphovascular invasion in the prognostic assessment of tumors.

On the other hand, the analysis of the association between peritumoral budding and lymph node status shows a lack of statistical significance in this group.

Peritumoral budding and perineural invasion (PNI):

The chi-square test results for the association between peritumoral budding and perineural invasion (PNI) are as follows:

Chi-square value: 5.59; *p*-value: 0.061; Df: 2.

These results suggest a marginally non-significant association, as the *p*-value is slightly above the standard threshold of 0.05. This indicates that while there may be some correlation between peritumoral budding and PNI, it does not achieve statistical significance.

#### 3.2.3. Important Prognostic and Predictive Biomarkers of Colorectal Carcinoma

##### MMR Status

MMR status and primary tumor localization:

1. dMMR (deficient mismatch repair): Most tumors (approximately 89%) in the dMMR group are localized in the right colon. This strong association suggests that dMMR status is more prevalent in right-sided colon cancer. There are very few left-sided tumors and no rectal tumors associated with dMMR, indicating a distinct localization pattern.
2. pMMR (proficient mismatch repair): Tumors in the pMMR group are more evenly distributed across all localization groups (right, left, and rectum), suggesting that pMMR tumors do not have a strong preference for a specific side. Rectal tumors are notably common in the pMMR group, accounting for nearly 35% of cases. This highlights a significant presence of rectal cancer in patients with proficient MMR. Left-sided tumors are also observed more frequently in the pMMR group compared with dMMR, reinforcing the variability in tumor location based on MMR status.

The chi-square test results confirm a statistically significant association between MMR status and tumor localization (right, left, and rectum):

Chi-square value: 20.28; *p*-value: 0.000039; Df: 2.

MMR status and sex:

For both sexes, the pattern for dMMR shows no statistically significant association between MMR status and sex.

MMR status and histological subtype:

The results for MMR status, histological subtype, and tumor localization are presented in the crosstab—Table 5.

The results of the chi-square test for statistical significance for MMR status vs. histological subtype and tumor localization are as follows:

Chi-square value: 36.72; *p*-value: 0.0467; Df: 24.

This test evaluates the combined influence of MMR status, histological subtype, and tumor localization. The *p*-value (0.0467) indicates a statistically significant association, but the strength of this association is less robust compared with separate tests due to a more complex model with higher degrees of freedom.

When we examined the relationship between MMR status and histological subtype separately, the following results from the chi-square test were obtained:

MMR status and histological subtype: chi-square value: 27.87; *p*-value: 0.001; Df: 9.

This indicates that there is a statistically significant association between MMR status and histological subtype.

MMR status and peritumoral stromal immune reaction, lymph node status, lymphovascular invasion, and perineural invasion:

The results indicated a lack of statistically significant associations for MMR status and peritumoral stromal immune reaction, lymph node status, lymphovascular invasion (LVI), and perineural invasion (PNI).

In Table 6, patients with dMMR and the corresponding MLH1, PMS2, MSH6, and MSH2 abnormalities identified through immunohistochemistry (IHC) are shown, along with the NGS-analyzed mutations in key genes and their combinations. In the last column, we indicate cases where further investigation for Lynch syndrome is warranted. In cases suspected of Lynch syndrome, no additional tests were conducted in our research to confirm the diagnosis. The research team declared a refusal to influence specific diagnostic or therapeutic decisions.

##### Gene Mutations Identified via NGS

In our study, we utilized the “TruSight Tumor 15” panel from Illumina for Next-Generation Sequencing (NGS). This panel is designed to detect mutations across 15 key genes associated with various cancers: AKT1, BRAF, EGFR, ERBB2(HER2), FOXL2, GNA11, GNAQ, KIT, KRAS, MET, NRAS, PDGFRA, PIK3CA, RET, and TP53.

The results obtained from NGS are as follows:

Observed mutations by sex:

1. Females: TP53 is the most frequent mutation, appearing in about 67.4% of cases. KRAS follows with a proportion of 34.9%. Other gene mutations include BRAF (18.6%), PIK3CA (9.3%), and NRAS (4.7%).
2. Males: TP53 is also the most common but slightly less frequent than in females, at 57.9%. KRAS appears in 38.6% of cases. Other gene mutations include PIK3CA (12.3%), BRAF (15.8%), and NRAS (3.5%).

Both sexes show a higher prevalence of TP53 mutations, but females have a slightly higher proportion. PIK3CA mutations appear more often in males compared with females.

The two-proportion Z-test for each gene mutation indicates no statistically significant differences in the proportions of any gene mutation between males and females.

**Figure 7:**
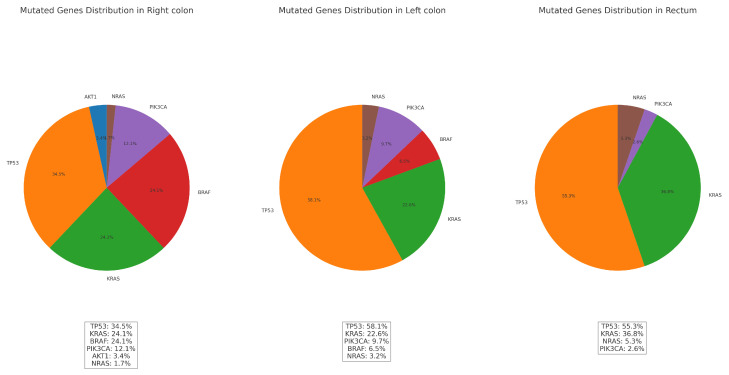
Mutated genes’ distribution by primary tumor localization—right colon, left colon, and rectum.

Observed mutations vs. primary tumor localization:

The distributions of gene mutations by count and frequency in relation to tumor localization are presented in Table 7 and Figure 7. These visualizations illustrate the variations in mutation prevalence across different tumor sites.

The results for the association between gene mutations and tumor localizations—chi-square value: 22.59; *p*-value: 0.012; Df: 10—indicate a statistically significant association between gene mutations and localization.

1. Right colon: Tumors located in the right colon tend to exhibit the highest number of mutations overall (42), with various gene mutations such as TP53, KRAS, BRAF, PIK3CA, and AKT1. BRAF and KRAS mutations are more frequent here compared with other locations. The high presence of mutations such as BRAF in the right colon aligns with known patterns in colorectal cancer and indicates a sporadic pathway rather than Lynch syndrome.
2. Left colon: The left colon tumors have fewer total mutations (25) than those in the right colon. The dominant mutations here include TP53 and KRAS, with PIK3CA also present but at a lower frequency. The mutations in the left colon tend to align more with classic genetic alterations seen in colorectal cancer. This area’s lower count of BRAF mutations differentiates it from the right colon.
3. Rectum: The rectal carcinomas have 29 total mutations, with a higher frequency of TP53 mutations. Unlike the right colon, the rectum shows no BRAF mutations, a crucial distinction. This suggests that the genetic profile of rectal cancers in this dataset differs significantly from right-sided colorectal cancers, which often exhibit BRAF mutations. The presence of KRAS and NRAS mutations indicates a different mutation spectrum in the rectum, which might be more associated with sporadic colorectal cancers rather than hereditary syndromes such as Lynch.

Mutations across tumor histological subtype:

The only gene mutation showing a significant association with histological type is BRAF. This suggests that the presence of BRAF mutations may vary depending on the histological subtype. The chi-square test results for BRAF mutations—chi-square value: 20.292; *p*-value: 0.016; Df: 9.

Mutations across tumor grade and stage:

1. The chi-square test for gene mutations in relation to tumor grade showed a statistically significant result only for BRAF mutations—chi-square: 12.070; *p*-value: 0.002; Df: 2. BRAF shows a statistically significant association with tumor grade. G2 is the grade with the highest frequency of mutations, particularly TP53 and KRAS. G3 shows TP53 as the most frequent mutation, with a slight increase in BRAF mutations. G4 has the fewest mutations overall.
2. The chi-square test for gene mutations related to tumor stages revealed a statistically significant association only with NRAS—chi-square: 11.929; *p*-value: 0.036; Df: 5. Stages pT1 and pT2: These early stages demonstrate limited mutations, with TP53 and KRAS mutations appearing occasionally. Stages pT3 and pT4: TP53 and KRAS mutations become more frequent at these advanced stages. Additionally, mutations in BRAF and PIK3CA are observed in some cases, indicating a shift toward a more aggressive tumor profile.

Mutations vs. lymph node status and lymphovascular invasion:

The results of the performed chi-square test indicate significant associations between specific gene mutations and both lymph node status and lymphovascular invasion in colorectal cancer:

TP53 Mutations: There is a notable association with both lymph node status and lymphovascular invasion. Chi-square: 7.423; *p*-value: 0.024; Df: 2.

BRAF Mutations: A significant association with lymphovascular invasion was also observed for BRAF mutations. Chi-square: 4.853; *p*-value: 0.028; Df: 1.

Analyses for gene mutations versus peritumoral budding, stromal immune reaction, and perineural invasion:

1. Peritumoral budding vs gene mutations:
  Bd1 (Bd0 + Bd1): TP53 and KRAS mutations are most frequent, with moderate occurrences of BRAF and PIK3CA.
  Bd2: Similar frequency for TP53, with KRAS and BRAF appearing less frequently.
  Bd3: Highest occurrence of TP53, followed by KRAS, BRAF, and some PIK3CA mutations.
  TP53 and peritumoral budding—chi-Square: 6.071; *p*-value: 0.048; Df: 2. This indicates a statistically significant association between TP53 mutations and peritumoral budding, suggesting that the presence of TP53 mutations may influence the tumor’s invasive characteristics, potentially impacting prognosis and treatment options.
2. Stromal immune reaction vs. gene mutations:
  Absent: TP53 and KRAS mutations are common, with minimal BRAF and PIK3CA.
  Moderate: TP53 and KRAS are frequent, with a presence of BRAF but fewer PIK3CA mutations.
  Marked: TP53 remains frequent, with higher occurrences of BRAF and PIK3CA.
  NRAS and stromal immune reaction—chi-square: 7.645; *p*-value: 0.022; Df: 2.
  Chi-square test for the associations reveals significant findings: The significant association between NRAS mutations and the stromal immune reaction indicates that NRAS may play a role in the immune landscape surrounding tumors, which could have implications for therapeutic strategies.
3. Perineural invasion vs. gene mutations:
  Negative: TP53 and KRAS mutations are most frequent; BRAF and PIK3CA appear less frequently.
  Positive: TP53 remains common, and there is an increase in PIK3CA mutations compared with negative cases.
  These results highlight the importance of genetic profiling in colorectal cancer, as understanding the relationships between specific mutations and clinical features can help inform prognosis and treatment decisions.

MMR status versus gene mutations:

Table 8 presents the results of the relationship between gene mutations and mismatch repair (MMR) status, highlighting a correlation between specific gene mutations and MMR status.

Only KRAS and BRAF mutations show significant associations with MMR status:

KRAS: Chi-square: 7.739; *p*-value: 0.005; Df: 1.

BRAF: Chi-square: 34.204; *p*-value: <0.001; Df: 1.

These results indicate a strong relationship between these specific mutations and the MMR status of colorectal tumors. The significant *p*-values suggest that KRAS and BRAF mutations are associated with particular MMR status groups, which may have implications for treatment decisions for individual genes and prognostic outcomes.

## 4. Discussion

In our study, we examined 100 patients, with a slight predominance of males—57 patients (57%) compared with 43 females (43%). This results in a male-to-female ratio of 1.33:1. The average age at diagnosis was 70 years. These findings align with global demographics for colorectal cancer (CRC), as the incidence of CRC tends to increase with age. Research conducted by Siegel et al., White et al., and Joo et al. supports our results, reporting similar median ages and sex distribution in CRC populations worldwide. [2,16,17].

Numerous studies have highlighted differences related to sex, age, and the location of primary tumors in various sections of the colon and rectum.

Based on research by Bufill et al.that examined the characteristics of colorectal carcinoma according to the embryonic origin of the right colon, left colon, and rectum, as well as findings from many subsequent studies, we categorized the different tumor locations into three groups: the right colon (cecum, ascending colon, hepatic flexure, and transverse colon), the left colon (splenic flexure, descending colon, and sigmoid colon), and the rectum.

Various studies have reported statistically significant differences in tumor location based on the sex and age of the patients [19,24].

Notably, the analysis revealed no statistically significant association between sex and tumor localization ($\chi^{2}$: 2.95; *p*-value: 0.938), which aligns with the findings of Kishiki et al., who also reported no gender-specific localization preferences [48].

In the group of patients we studied, right-sided colon cancers were found to be more common, a finding that aligns with results reported by other researchers [20,24,49].

However, our study did not reveal statistically significant differences in tumor localization among the right colon, left colon, and rectum when considering gender and age.

The statistical results on tumor localization, histological subtype, tumor grade, and stage showed statistically significant differences when comparing tumor differentiation and stage. Most tumors are classified as moderate grade (G2), with advanced stages (T3/T4).

In evaluating important histological tumor characteristics such as lymphatic and vascular invasion, perineural invasion and tumor localization, tumor grade, and stage, a significant correlation exists between tumor grade and lymphovascular invasion (LVI) ($\chi^{2}$: 17.33; *p*-value: 0.00017), supporting the hypothesis that higher grades are associated with more aggressive tumor behavior. These results align with studies conducted by Saito et al., who also found higher rates of LVI among advanced CRC grades [38].

LVI was observed in 26% of cases, demonstrating a significant association with higher tumor grades (*p* < 0.05). This aligns with the research conducted by Des Guetz et al., who recognized LVI as a reliable marker for predicting poor prognosis and increased metastatic risk in colorectal cancer patients [14].

However, perineural invasion (PNI) did not exhibit statistically significant correlations with tumor grade, stage, or localization (all *p*-values > 0.05). This outcome reflects findings by Lee et al., which reported inconsistent prognostic value for PNI across CRC cases [24].

When evaluating the Crohn’s-like stromal immune response in colorectal carcinoma based on its localization in the right colon, left colon, and rectum, we found statistically significant associations. The immune response was more pronounced in the right colon, which aligns with the significant data regarding the prevalence of dMMR tumors in the same area.

The interaction between the peritumoral stromal immune response and various tumor characteristics, including histological subtype, grade, stage, nodal status, lymphovascular invasion, and perineural invasion, did not reveal statistically significant associations.

This study identified no significant relationship between tumor budding and overall stage (*p* > 0.05), mirroring findings by Benedix et al., who noted that the impact of tumor budding varies according to additional factors, including tumor localization and genetic mutations [19].

Tumor budding has been significantly correlated with higher tumor grades within this cohort, thereby validating its role as a marker of aggressive tumor behavior. Research conducted by Lugli et al. has identified similar patterns, emphasizing the association between tumor budding and unfavorable clinical outcomes [37].

A statistically significant association exists between peritumoral budding and lymphovascular invasion, while no significant association is observed between tumor budding and nodal status.

The absence of a statistically significant association between tumor budding and lymph node status (*p*-value: 0.132) suggests that multiple histological and molecular factors, beyond peritumoral budding, contribute to lymph node involvement.

A statistically significant correlation between MMR status and tumor localization—right, left, and rectum, ($\chi^{2}$: 20.28; *p*-value: 0.000039) confirms the distinct biological behavior observed in right-sided colorectal cancers. This aligns with previous observations that right-sided tumors usually display unique molecular profiles, including a status of deficient mismatch repair (dMMR). The study shows that 89% of dMMR cases occur in right-sided tumors, reinforcing the findings of Yahagi et al. that highlight the prevalence of genetic anomalies, such as BRAF mutations, in this particular tumor location [23].

Genetic analysis revealed several key mutations, including KRAS, BRAF, and TP53. The majority of BRAF mutations were identified in right-sided tumors, which aligns with findings from the literature on sporadic colorectal cancer cases. Furthermore, dMMR was prevalent in 89% of right-sided cases, reinforcing its role as a biomarker for this subtype. This corresponds with the findings of Boland and Goel, who affirm the diagnostic and therapeutic significance of microsatellite instability (MSI) and MMR status in the management of colorectal cancer [13,50].

The prognostic significance of specific mutations—particularly KRAS, BRAF, and TP53—plays a crucial role in understanding cancer outcomes. KRAS mutations are detectable across various tumor stages and anatomical locations. They are known to contribute to resistance against anti-EGFR therapies, highlighting the need for alternative treatment strategies. BRAF mutations, which are predominantly found in right-sided colorectal cancers, are linked to poor prognostic outcomes and require targeted therapeutic approaches. These findings align with the research presented by Venderbosch et al. [51]. Additionally, the presence of BRAF mutations in colorectal cancer (CRC) is associated with a higher likelihood of metastatic disease, indicating the aggressive nature of this cancer subtype. Research conducted by Domingo et al. highlights that BRAF-mutated CRC is characterized by distinct biological behaviors and poorer prognostic outcomes [42]. Such mutations are frequently associated with more advanced-stage diagnoses and a heightened propensity for early metastasis, particularly to the liver and peritoneum. TP53 mutations are commonly observed across various stages and localizations within colorectal cancer, highlighting their significant role in tumor progression. These mutations contribute to CRC’s molecular complexity and heterogeneity, influencing tumors’ biological behavior and responses to therapeutic interventions [52].

## 5. Conclusions

This study focuses on the diversity of colorectal cancer (CRC) by examining how genetic and histological characteristics vary based on tumor location or other tumor variables. It highlights the importance of biomarkers such as mismatch repair (MMR) status, tumor budding, BRAF mutations, and immune responses in guiding personalized treatments for CRC. However, the limited sample size restricted the ability to identify subtle associations. The authors emphasize the need for further comprehensive studies in CRC tumor pathology.

## Figures and Tables

**Table 1:** The median age of patients relative to tumor localization in the overall patient group differentiated by sex and age ranges. Statistical analysis indicates no statistically significant differences in the distribution by sex ($\chi^{2}$: 2.95; *p*-value: 0.938) or age (Kruskal–Wallis test statistic is approximately 3.25, *p*-value: 0.777) in relation to different tumor localizations.

	Median Age	Range	Patients	Median Males	Range Males	Total Males	Median Females	Range Females	Total Females
Ascendens	70.5	55–88	14	71.0	67–88	7	61.0	55–75	7
Cecum	71.5	56–86	16	71.5	57–86	10	71.5	56–83	6
Descendens	75.0	48–81	6	76.0	57–81	4	61.5	48–75	2
Fl. hepatica	68.0	68–68	1	68.0	68–68	1			
Fl. lienalis	69.5	61–78	4	71.0	68–78	3	61.0	61–61	1
Rectum	71.0	45–86	29	72.5	45–86	16	68.0	57–77	13
Sigmoideum	68.0	48–77	19	68.0	57–77	9	68.5	48–76	10
Transversum	65.0	57–84	11	65.0	57–84	7	73.0	61–75	4
Total	70.0	45–88	100	70.0	45–88	57	70.0	48–83	43

**Table 2:** Chi-square test results for LVI and tumor Grade and Stage.

Chi-Square Value	Grade	Stage
$\chi^{2}$ value	17.33	16.13
Df	2	5
*p*-value	0.00017	0.006

**Table 3:** Results of the chi-square tests for the association between perineural invasion (PNI) and localization, histological subtype, grade, and stage.

	Localization	Histological Subtype	Grade	Stage
$\chi^{2}$ value	1.46	7.31	2.15	2.46
*p*-value	0.984	0.605	0.542	0.783
Df	7	9	3	5

**Table 4:** Results for tumor grade and tumor stage and peritumoral budding—Bd1 (Bd0 + Bd1), Bd2, Bd3.

Tumor Grade	Bd1 (Patient Count)	Bd2 (Patient Count)	Bd3 (Patient Count)
G2	35	3	1
G3 + G3 NEC	29	1	0
G4	21	8	2
Tumor stage			
T1	1	0	1
T2	7	5	6
T3	24	22	18
T4	4	1	3
T4A	1	2	3
T4B	2	0	0

**Table 5:** MMR status, histological subtype, and primary tumor localization: All cases of dMMR-associated adenocarcinoma NOS, medullary tumors, MiNEN, and the single case of serrated adenocarcinoma were located in the right colon. Most dMMR-associated mucinous adenocarcinomas (four out of six) were found in the right colon, with two in the left colon; no dMMR-associated tumors were found in the rectum.

MMR Status	Histological Type	Right	Left	Rectum
dMMR	Adenocarcinoma NOS	8	0	0
	Medullary	2	0	0
	MiNEN	1	0	0
	Mucinous	4	2	0
	Serrated adenocarcinoma	1	0	0
pMMR	Adenocarcinoma NOS + mucinous component	0	0	1
	Adenocarcinoma NOS + intra and extracellular mucin production	0	0	1
	Adenocarcinoma NOS	21	22	26
	Adenocarcinoma NOS with mucinous and poorly cohesive component	1	0	0
	Mucinous	2	3	0
	Serrated adenocarcinoma	1	1	1
	Synchronous cecum (mucinous) + rectum (adenocarcinoma NOS)	1	0	0
	Undifferentiated	0	1	0

**Table 6:** dMMR status with corresponding MLH1, PMS2, MSH6, and MSH2 status; main gene mutations; and mutated gene combinations and their relation to Lynch syndrome.

Patient	MMR	MLH1	PMS2	MSH6	MSH2	BRAF Gene Mutations	Mutated Genes and Combinations	Suspicion for Lynch Syndrome
10	dMMR	neg	neg	pos	pos	BRAF	BRAF + TP53	Not Suspected
16	dMMR	neg	neg	pos	pos	BRAF	BRAF + TP53	Not Suspected
19	dMMR	neg	neg	neg	pos	BRAF	BRAF + TP53	Not Suspected
20	dMMR	neg	neg	pos	pos	BRAF	BRAF + TP53	Not Suspected
33	dMMR	neg	neg	pos	pos	BRAF	BRAF	Not Suspected
52	dMMR	neg	neg	pos	pos	BRAF	BRAF + PIK3CA	Not Suspected
56	dMMR	neg	neg	pos	pos	BRAF	BRAF	Not Suspected
58	dMMR	neg	neg	pos	pos	BRAF	BRAF	Not Suspected
60	dMMR	neg	neg	pos	pos		TP53	Suspected
65	dMMR	pos	pos	neg	neg		No Result	Suspected
67	dMMR	pos	pos	neg	pos		PIK3CA + TP53 + KRAS	Suspected
75	dMMR	neg	pos	pos	neg		No Result	Suspected
84	dMMR	neg	neg	pos	pos	BRAF	BRAF	Not Suspected
88	dMMR	neg	neg	pos	pos	BRAF	BRAF + AKT1	Not Suspected
92	dMMR	neg	neg	pos	pos		NRAS + TP53	Suspected
94	dMMR	neg	neg	pos	pos	BRAF	BRAF + PIK3CA	Not Suspected
95	dMMR	neg	neg	pos	pos	BRAF	BRAF + TP53	Not Suspected
96	dMMR	neg	neg	pos	pos		PIK3CA	Suspected

**Table 7:** Mutations and primary tumor localization. (For 12 of the studied patients, there is a lack of data regarding the mutational profile due to insufficient material, technical reasons, and other factors).

Localization	Total	AKT1	TP53	KRAS	BRAF	PIK3CA	NRAS
Right colon	42	2	20	14	14	7	1
Left colon	25	0	18	7	2	3	1
Rectum	29	0	21	14	0	1	2

**Table 8:** MMR status in relation to the mutations.

MMR Status	AKT1	TP53	KRAS	BRAF	PIK3CA	NRAS
dMMR	1	8	1	12	4	1
pMMR	1	54	36	5	7	3

## Data Availability

The data from this study are available on the Science Data Bank website. Interested parties may access the data by submitting a request to the authors of the study.

## Abbreviations

The following abbreviations are used in this manuscript:

CRC	Colorectal cancer
PNI	Perineural invasion
LVI	Lymphovascular invasion
Bd	Budding
MMR	Mismatch repair
pMMR	Proficient mismatch repair
dMMR	Deficient mismatch repair
NGS	Next-Generation Sequencing
Fl. hepatica	Hepatic flexure
Fl. lienalis	Splenic flexure
NOS	Not otherwise specified
MiNEN	Mixed neuroendocrine-non neuroendocrine neoplasms
FFPE	Formalin-fixed paraffin-embedded
Df	Degrees of freedom
ITBCC	International Tumor Budding Consensus Conference
IHC	Immunohistochemistry
